# Longitudinal change in energy expenditure and effects on energy requirements of the elderly

**DOI:** 10.1186/1475-2891-12-73

**Published:** 2013-06-06

**Authors:** Jamie A Cooper, Todd M Manini, Chad M Paton, Yosuke Yamada, James E Everhart, Steve Cummings, Dawn C Mackey, Anne B Newman, Nancy W Glynn, Fran Tylavsky, Tamara Harris, Dale A Schoeller

**Affiliations:** 1 Department of Nutritional Sciences, Hospitality, and Retailing, Texas Tech University, PO Box 41240, Lubbock, TX 79409, USA; 2 Department of Aging and Geriatric Research, University of Florida, 2199 Mowry Road, Building 2020, Gainesville, FL, 32611, USA; 3 Laboratory of Sports and Health Science, Kyoto Prefectural University of Medicine, 465 Kajii-cho, Kamigyo-ku, Kyoto, 602-8566, Japan; 4 The Japan Society for the Promotion of Science (JSPS) research fellow (SPD), 5-3-1 Kojimachi, Chiyoda-ku, Tokyo, 102-0083, Japan; 5 Epidemiology and Clinical Trials Branch, NIH NIDDK, 2 Democracy Plaza, room 655, Bethesda, MD, 20892-5450, USA; 6 San Francisco Coordinating Center, California Pacific Medical Center Research Institute, San Francisco, CA, USA; 7 Department of Epidemiology, Center for Aging and Population Health, University of Pittsburgh, 130 N. Bellefield Ave, Pittsburgh, PA, 15213, USA; 8 Department of Epidemiology, Center for Aging and Population Health, University of Pittsburgh, 130 DeSoto Street, Pittsburgh, PA, 15261, USA; 9 Department of Preventive Medicine, University of Tennessee-Memphis, 66 North Pauline Street, Suite 633, Memphis, TN, 38105, USA; 10 National Institute on Aging, National Institutes of Health, Building 31, Room 5C27 31 Center Drive, MSC 2292, Bethesda, MD, 20892, USA; 11 Department of Nutritional Sciences, University of Wisconsin-Madison, 1415 Linden St., Madison, WI, 53706, USA

**Keywords:** Doubly labeled water, Energy expenditure, Elderly, Energy requirements

## Abstract

**Background:**

Very little is known about the longitudinal changes in energy requirements in late life. The purposes of this study were to: (1) determine the energy requirements in late life and how they changed during a 7 year time-span, (2) determine whether changes in fat free mass (FFM) were related to changes in resting metabolic rate (RMR), and (3) determine the accuracy of predicted total energy expenditure (TEE) to measured TEE.

**Methods:**

TEE was assessed via doubly labeled water (DLW) technique in older adults in both 1999 (n = 302; age: 74 ± 2.9 yrs) and again in 2006 (n = 87 age: 82 ± 3.1 yrs). RMR was measured with indirect calorimetry, and body composition was assessed with dual-energy x-ray absorptiometry.

**Results:**

The energy requirements in the 9th decade of life were 2208 ± 376 kcal/d for men and 1814 ± 337 kcal/d for women. This was a significant decrease from the energy requirements in the 8th decade of life in men (2482 ± 476 kcal/d vs. 2208 ± 376 kcal/d) but not in women (1892 ± 271 kcal/d vs. 1814 ± 337 kcal/d). In addition to TEE, RMR, and activity EE (AEE) also decreased in men, but not women, while FFM decreased in both men and women. The changes in FFM were correlated with changes in RMR for men (r = 0.49, p < 0.05) but not for women (r = −0.08, ns). Measured TEE was similar to Dietary Reference Intake (DRI) predicted TEE for men (2208 ± 56 vs. 2305 ± 35 kcal/d) and women (1814 ± 42 vs. 1781 ± 20 kcal/d). However, measured TEE was different than the World Health Organization (WHO) predicted TEE in men (2208 ± 56 vs. 2915 ± 31 kcal/d (p < 0.05)) and women (1814 ± 42 vs. 2315 ± 21 kcal/d (p < 0.05)).

**Conclusions:**

TEE, RMR and AEE decreased in men, but not women, from the 8th to 9th decade of life. The DRI equation to predict TEE was comparable to measured TEE, while the WHO equation over-predicted TEE in our elderly population.

## Background

Accurate estimates of energy requirements are vital because they form the platform to which all other required nutrients must be affixed. Too high of an estimate may encourage excessive energy intake and weight gain, while the opposite can lead to weight loss [1]. This is an important issue since more than one-third of all U.S. adults are considered obese [2]. Further, over the past 30 years, the proportion of older adults who are obese has doubled and more than 70% of people over the age of 65 are overweight or obese [3]. This is problematic since obesity has been shown to be an independent risk factor for developing CVD [4]. The issue of obesity is especially important in an elderly population as CVD remains the leading cause of morbidity and mortality in U.S. adults with 84% of people over the age of 65 dying from CVD [5]. Therefore, understanding energy requirements and how they change over time in the elderly can be extremely important and clinically relevant for both the prevention of weight gain and reducing the risk of developing chronic diseases.

Energy requirements, however, have historically been hard to estimate and for many decades were based on imprecise estimates of energy intake. During the 1980s, the World Health Organization (WHO) proposed that better estimates of energy requirements should be obtained from measurements of total energy expenditure (TEE) [6]. About this same time, the first application of the doubly labeled water (DLW) technique to measure TEE appeared [7]. DLW is the most accurate and precise method for measuring free-living TEE [8] and now forms the basis for most estimates of energy requirements.

In the current Dietary Recommended Intakes (DRI), it is noted that TEE declines progressively with increasing age (approximately 150kcals per decade) and this appears to be linear across the adult life span [9]. Three factors make up TEE: resting metabolic rate (RMR), the thermic effect of feeding (TEF), and activity energy expenditure (AEE). While some of the data regarding the TEF are conflicting, most studies show that this component of TEE does not decline with aging [9]. The two factors that primarily account for that decline in TEE are resting metabolic rate (RMR) and AEE [10-12]. The reduction in RMR is due primarily to losses in fat-free mass (FFM) and gains in less metabolically active fat tissue [9]. Levels of AEE decline rapidly after the 5th decade of life; however, small sample sizes in studies makes it difficult to determine the rate of decline [9]. Both the decreases in TEE and AEE not only increase obesity risk in elderly individuals, but are also accompanied by a decreased ability to perform activities of daily living such as eating, dressing, and general mobility [13].

Some equations have been created to predict TEE such as the DRI and WHO equations [6,14]. However, the accuracy of these equations in individuals who are in their 9th decade of life remains unknown. DLW has been used previously to determine free-living energy expenditure (EE) in elderly adults [11,12,15,16]. However, most of these studies are carried out on people in their 7th or 8th decade of life. These data are consistent with a decline in TEE with age, but the data on energy requirements for subjects >80 years of age is sparse [17]. Moreover, these data are derived from cross-sectional studies and thus observed changes seen with aging may include cohort effects. The Health, Aging, and Body Composition (Health ABC) study has measures of TEE from DLW in elderly men and women (8th decade of life) [18]. In order to characterize the longitudinal change in components of TEE to help better estimate the changes in energy requirements during aging, DLW measures of TEE were also measured 7 years later (9th decade of life). The purposes of this study were 3-fold: (1) to determine the energy requirements in very late life and how those requirements changed during a 7 year time-span for both men and women, (2) to determine whether changes in body composition (FFM) were related to changes in RMR, and (3) to determine the accuracy of predicted TEE to measured TEE in an elderly population and what the best predictors of TEE are.

## Method

### Subjects

In 1997–1998, investigators from the University of Pittsburgh and University of Tennessee, Memphis, recruited 3075 participants ages 70–79 years from a random sample of white Medicare beneficiaries and all age eligible self-identified black community residents to participate in the Health, Aging, and Body Composition (Health ABC) study. To be eligible, participants had to be able to walk 0.25 miles (400 m), climb 10 stairs, and perform activities of daily living. They also had to have no plans to leave the area for the next 3 years and have no evidence of a life-threatening illness. The sample was approximately balanced for sex (51% women; 49% men), and 42% of the participants were black. Written informed consent and approval by the institutional review boards at both the University of Pittsburgh and University of Tennessee, Memphis, was obtained from each individual prior to participation in the study.

An energy expenditure (EE) component (baseline - visit 1) was carried out between 1998 and 2000. A randomly selected list of 500 participants stratified by race and sex was generated from study-eligible individuals which included those who (1) did not have a recent blood transfusion, (2) did not use supplemental oxygen or insulin, and (3) did not plan overnight travel immediately before or during the EE study. A total of 323 participants were enrolled in the EE component (n = 92 in 1998, n = 125 in 1999, and n = 85 in 2000). Twenty-one participants were excluded from the analysis due to failure to complete the study protocol, lack of adequate urine volume specimens, or failure of isotope (missing data points, insufficient dose recovery, or problems during analysis) or RMR data to meet a priori quality-control criteria. This left 302 participants (n = 150 men and 152 women) for the final analysis. Compared with the full Health ABC cohort, there were no differences in age, sex, body mass, fat free mass (FFM), fat mass (FM), gait speed, self-reported walking ability, or self-reported physical activity (walking, stair climbing, working, volunteering, and caregiving).

A follow-up EE component was carried out in 2006 (follow-up - visit 2), approximately 7 years after the baseline EE component. Those individuals who had participated in the baseline EE were invited to complete the follow-up EE component. The same inclusion/exclusion criteria for baseline EE was applied to the follow-up EE. Of the 302 participants that had both TEE and RMR data for baseline, only 104 were eligible and interested in the follow-up (66 had died before the 2006 follow-up, 1 withdraw, and 131 were not eligible or interested). Of those 104 participants who were eligible and interested, 17 were excluded based on missing a study visit, having a blood transfusion within 7 days of the DLW study visit, or having unacceptable or missing TEE or RMR data. Therefore, only 87 participants (47 men and 40 women) had both TEE and RMR data from for both baseline and follow-up. Compared to the full Health ABC study participants, there were no differences in age, sex, body mass, fat free mass (FFM), fat mass (FM), gait speed, self-reported walking ability, or self-reported physical activity (walking, stair climbing, working, volunteering, and care-giving) with the subjects in the follow-up EE study.

### Protocol

The protocol was similar for both cohorts. Participants completed the protocol over 2 visits to the clinic, each time arriving in a fasted state. During visit 1, participants received a dose of DLW for measurement of TEE according to a protocol previously described [16,19] and body composition was measured with dual-energy X-ray absorptiometry (DXA). Participants then returned to the clinic for a second visit 14 ± 1 day after visit 1 where body weight and RMR were measured. Additionally, two urine samples were collected for DLW analysis. Participants were encouraged to maintain their normal activity levels between visits 1 and 2.

### Total energy expenditure (TEE)

TEE was measured in the same manner in both cohorts by using the 2-point DLW technique which has previously been described in detail [19]. Briefly, during visit 1, participants provided a baseline urine sample and then ingested 2 g/kg estimated total body water (TBW) of DLW, which was composed of 1.9 g/kg estimated TBW (10% H_2_^18^O) and 0.12 g/kg estimated TBW (99.9% ^2^H_2_O). Urine samples were then collected 2, 3, and 4 hours after dosing. Approximately 14 days later at visit 2, two more urine samples were obtained. Plasma from a 5-mL blood sample was obtained from everyone, but was only used for those who had evidence of delayed isotopic equilibration likely caused from urine retention in the bladder (n = 28) [19]. Urine and plasma samples were stored at −20°C until analyzed by isotope ratio mass spectrometry.

Dilution spaces for ^2^H and ^18^O were calculated according to Coward [20]. TBW was calculated as the average of the dilution spaces for ^2^H and ^18^O after correction for isotopic exchange (1.041 for ^2^H and 1.007 for ^18^O). Carbon dioxide production was calculated by using the 2-point DLW method outlined by Schoeller et al. [8,21], and TEE was derived with the Weir equation [22] and a food quotient of 0.86 was used [23,24]. All EE values were converted to kilocalories per day, and the thermic effect of meals was assumed to be 10% of TEE [25]. For measurement of TBW, the intra-subject repeatability (calculated as the average percentage difference between the 2 analyses) was −0.1 ± 1.2%. The intra-tester repeatability of TEE based on blinded, repeat, urine isotopic analysis was 1.2 ± 5.4% (n = 16) and compared well with recent literature [10].

### Resting metabolic rate (RMR)

RMR was calculated using indirect calorimetry on the Deltatrac II respiratory gas analyzer (Datex Ohmeda Inc, Helsinki, Finland) with participants in the fasted state after a 30-minute rest and has been described in detail elsewhere [16]. Briefly, respiratory gas exchange was measured minute-by-minute for 40 minutes with only the last 30 minutes used in RMR calculations. Movement or sleeping during the test was noted and those time periods were excluded from the calculation. Methanol burn tests were performed in duplicate once or twice a month. Carbon dioxide recovery averaged 100.1 ± 1.4% at the Pittsburgh site and 100.5 ± 1.5% at the Memphis site. The respiratory exchange ratios for methanol differed by 2.5% between sites (Memphis: 0.66 ± 0.01; Pittsburgh: 0.68 ± 0.001, p < 0.001), and this difference did not demonstrate a trend over time. Therefore, a correction factor was used to equate the 2 study sites by dividing the respiratory ratios for participants enrolled at Pittsburgh by 1.025. To calculate an adjusted RMR, RMR was divided by FFM. Additionally, physical activity level (PAL) was calculated as TEE/RMR and AEE was calculated as TEE*0.9-RMR.

### Body mass and composition

Total body mass, FM, and FFM were measured by DXA with a Hologic 4500A Scanner (Hologic Inc, Waltham, MA). Body composition analysis from DXA was performed with the Hologic software (version 8.21; Hologic Inc). Calibration was performed 3 times per week by using whole-body quality control phantoms outlines in the Hologic manual. Absolute variation between the clinic sites was monitored by cross-calibrating the 2 scanners with the use of separate phantoms. Validation on the scanners detected a systematic overestimation of FFM that was subsequently corrected by multiplying by a factor of 0.964 (see reference [26] for more details). FFM was calculated after removing mass due to bone mineral content (BMC). Finally, other measurements such as blood pressure and medical conditions including coronary heart disease, cerebrovascular disease, cardiovascular disease, diabetes, any type of cancer, and osteoporosis were only recorded at baseline.

### DRI and WHO energy requirement estimates

Energy requirements for individuals in very late life were calculated using data from the follow-up (height, weight, age, and sex) with both the WHO and DRI equations. These estimated energy requirements were calculated for comparison with the DLW measured TEE. To determine energy requirements using the WHO equations, we used the equation designed for men age 60 and women ages 31–60 since no equations exist for individuals over the age of 60. Further, the lowest activity factor (1.6) was used for the WHO equation in both men and women. For the DRI equations, the “low active” physical activity (PA) factor was used for men (PA of 1.11) and for women (PA of 1.12).

### Data analysis

SAS version 9.2 statistical package (SAS Institute Inc, Cary, NC) was used for all data analysis. Comparisons between men and women for study variables were performed with independent samples t-tests at both baseline and follow-up. Outcome variables (TEE, RMR, adjusted RMR, AEE, PAL, height, weight, BMI, body fat percentage, and FFM) by sex from baseline to follow-up were analyzed with a paired *t*-test. Pearson correlations were used to examine changes in RMR with changes in FFM from baseline to follow-up. Further, an ANOVA was used to compare DLW measured TEE with both the WHO and DRI predicted TEE. Finally, a step-wise regression analysis was performed to determine predictors of TEE in the elderly. Data are presented as mean ± SD unless otherwise indicated, and statistical significance was set at p < 0.05.

## Results

Subject characteristics of the study sample for all of the subjects that had DLW measurements in 2006 (follow-up) are shown in Table 1. Significant sex differences existed for TEE, RMR, adjusted RMR, AEE, height, body weight, FFM, and body fat percentage. Men had a higher TEE, RMR, AEE, height, body weight, and FFM and had a lower body fat percentage and adjusted RMR compared to women. Total EE in the 8th decade of life for men was 2249 ± 413 kcal/d and 1781 ± 315 kcal/d for women. Compared to those who did not participate, the follow-up sample had a lower prevalence of cancer (n = 10 vs. n = 23 for those lost to follow-up), lower prevalence of CVD (n = 18 vs. n = 58 for those lost to follow-up), lower prevalence of diabetes (n = 10 vs. n = 29 for those lost to follow-up), lower prevalence of osteoarthritis (n = 7 vs. n = 22 for those lost to follow-up), and a lower prevalence of osteoporosis (n = 8 vs. n = 25 for those lost to follow-up). Therefore, this follow-up sample was biased toward those individuals healthy enough to participate in the follow-up study.

**Table 1:** Characteristics of the study sample

	**2006**	
	**Men**	**Women**
	**n = 59**	**n = 55**
**Metabolic Parameters**		
Total energy expenditure (kcal/day)	2249 ± 413^*^	1781 ± 315
Resting metabolic rate (kcal/day)	1319 ± 182^*^	1085 ± 141
Resting metabolic rate adjusted for lean mass (RMR/FFM) (kcal/day)	26.1 ± 2.4^*^	28.7 ± 2.3
AEE (TEE*0.9-RMR) (kcal/day)	723 ± 239^*^	578 ± 251
PAL (TEE/RMR)	1.70 ± 0.23	1.65 ± 0.28
**Demographics**		
Age, M (SD)	82.2 ± 3.2	82.0 ± 3.1
Height (cm)	170.9 ± 6.4^*^	157.3 ± 6.7
Weight (kg)	79.5 ± 13.8^*^	68.2 ± 11.2
Fat Free Mass (kg)	52.4 ± 6.6^*^	39.3 ± 5.0
BMI (kg/m^2^)	27.2 ± 4.6	27.6 ± 4.6
Body Fat Percentage (DXA)	28.8 ± 6.0^*^	39.6 ± 4.7
Blood Pressure (S = Systolic; D = Diastolic) mmHg	S:135 ± 17 D:73 ± 10	S:142 ± 22 D:71 ± 10
Memphis Site, %	53.3%	54.6%

^* ^= significant difference between men and women.

Data presented as Mean ± Standard Deviation.

Comparisons in sex differences for each variable, as well as the changes in each variable, for both baseline and follow-up are presented in Table 2. This was only done in subjects who had DLW measures of TEE in both visits. Therefore, the sample size for the change in variables was much smaller (n = 47 for men and n = 40 for women). Further, because there were fewer subjects that had DLW measures both for baseline and follow-up, the subject characteristics in Table 1 differ slightly from Table 2 for follow-up 2 participants. From 1999 to 2006, men showed significant decreases in TEE, RMR, AEE, PAL, height, body weight, and FFM. No differences were found for adjusted RMR (RMR/FFM), BMI, or body fat percentage. Similarly, women had significant decreases in height, body weight, FFM, and body fat percentage. However, there were no significant changes in TEE, adjusted RMR, AEE, PAL, or BMI. There was a trend toward a decrease in RMR (p = 0.06). Based on the TEE data, energy requirements decreased significantly in men from visit 1 (2482 ± 476 kcal/d) to visit 2 (2208 ± 376 kcal/d) while they did not change in women from visit 1 (1892 ± 271 kcal/d) to visit 2 (1814 ± 337 kcal/d).

**Table 2:** **Measures of subjects with doubly labeled water data in *both *1999 and 2006**

	**1999**	**2006**	**1999**	**2006**
	**Men**	**Men**	**Women**	**Women**
	**n = 47**	**n = 47**	**n = 40**	**n = 40**
**Metabolic Parameters**				
Total energy expenditure (kcal/day)	2482 ± 476^a, c^	2208 ± 376^b^	1892 ± 271	1814 ± 337
Resting metabolic rate (kcal/day)	1401 ± 204^a, c^	1322 ± 182^b^	1133 ± 157^d^	1097 ± 148
Resting metabolic rate adjusted for mass (RMR/FFM) (kcal/day)	25.8 ± 2.6^a^	25.5 ± 2.1^b^	28.0 ± 3.0	27.6 ± 2.9
AEE (TEE*0.9-RMR) (kcal/day)	832 ± 308^a, c^	666 ± 243^b^	568 ± 181	540 ± 277
PAL (TEE/RMR)	1.77 ± 0.23^a, c^	1.68 ± 0.21	1.68 ± 0.19	1.67 ± 0.31
**Demographics**				
Age, M (SD)	74.7 ± 3.2^c^	82.2 ± 3.3	74.5 ± 2.8^c^	82.0 ± 2.8
Height (cm)	172.8 ± 6.9^a, c^	170.6 ± 6.8^b^	159.3 ± 6.5^c^	157.1 ± 6.6
Weight (kg)	80.6 ± 12.3^a, c^	78.7 ± 13.5^b^	72.0 ± 11.1^c^	68.9 ± 10.6
Fat Free Mass (kg)	54.5 ± 7.6^a, c^	52.0 ± 6.6^b^	40.6 ± 4.6^c^	39.9 ± 5.0
BMI (kg/m^2^)	27.0 ± 4.3	27.1 ± 4.8	28.4 ± 4.5	28.0 ± 4.3
Body Fat Percentage (DXA)	28.5 ± 5.5^a^	28.9 ± 6.3^b^	41.3 ± 4.2^c^	39.6 ± 4.4

^a ^= significant difference between men and women in 1999.

^b ^= significant difference between men and women in 2006.

^c ^= significant difference between 1999 and 2006 data for either men or women.

^d ^= trend for a significant difference between 1999 and 2006 at p = 0.06.

Data presented as Mean ± Standard Deviation.

**Figure 1:**
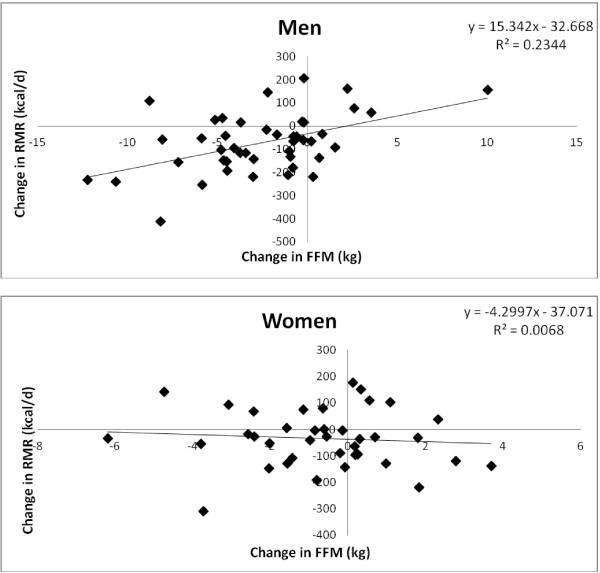
**Changes in resting metabolic rate versus changes in fat free mass. **Correlation between the changes in FFM and RMR in both men and women from visit 1 (1999) to visit 2 (2006). The change in RMR was positively correlated with the change in FFM for men (r = 0.49, p < 0.001) but not for women (r = −0.08, ns). FFM = Fat Free Mass. RMR = Resting Metabolic Rate.

The correlation coefficients between the change in RMR over the seven years vs. the change in FFM was calculated in both men and women (Figure 1). The change in RMR was positively correlated with the change in FFM for men (r = 0.49, p < 0.001) but not for women (r = −0.08, ns). Since TEE only changed significantly in men, we also examined correlations between changes in TEE with either changes in FFM or RMR. Changes in FFM were not correlated with changes in TEE (r = 0.22, ns), but there was a trend between changes in TEE with changes in RMR (r = 0.27, p = 0.07) in men.

**Figure 2:**
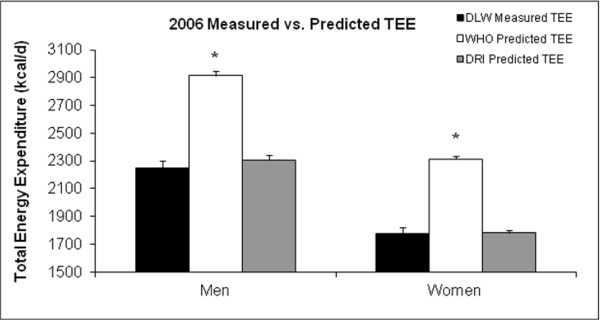
**Measured versus predicted total energy expenditure in visit 2. **Measured TEE from DLW in the 2006 visit versus predicted TEE from the WHO predicted TEE equation and the DRI predicted TEE equation. The WHO predicted TEE was significantly higher than the measured TEE from DLW (p < 0.05). No differences were found between measured TEE and the DRI predicted TEE. Data presented as Mean ± SEE. * Denotes significant difference between measured and predicted TEE at p < 0.05. DLW = Doubly labeled water. DRI = Dietary Reference Intake. TEE = Total energy expenditure. WHO = World Health Organization.

**Figure 3:**
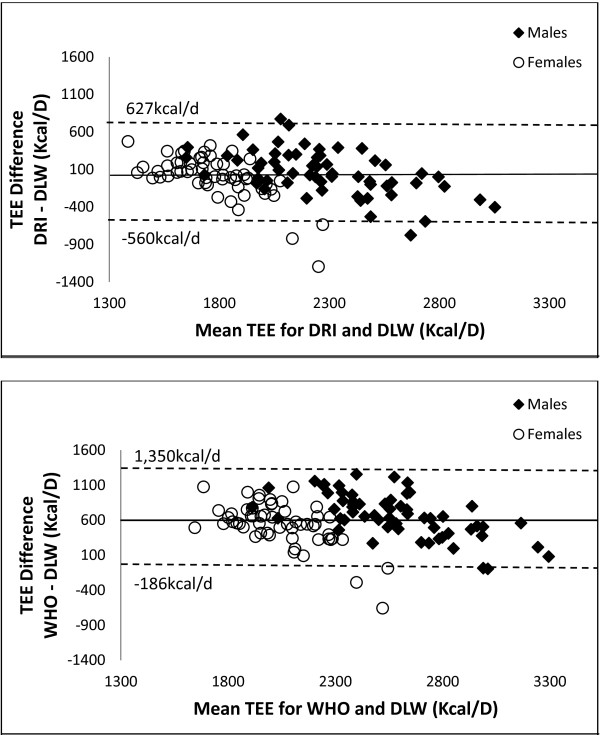
**Bland altman plots for measured and predicted TEE. **Bland-Altman plots of TEE for the DRI and the WHO compared to measured TEE with DLW. Straight lines represent the average TEE difference; dashed lines represent ±2 standard deviations. DLW = Doubly labeled water. DRI = Dietary Reference Intake. TEE = Total energy expenditure. WHO = World Health Organization.

The estimated energy requirements calculated with the WHO and DRI equations were compared with the DLW measured TEE (Figure 2). We also created Bland-Altman plots to compare predicted TEE with DLW measured TEE. Figure 3 shows the Bland-Altman plot between DRI predicted TEE and DLW measured TEE (limits of agreement: -560 to 627 kcals/D) and the plot between WHO predicted TEE and DLW measured TEE (limits of agreement: -186 to 1,350 kcals/D). There was a significant difference between the DLW measured TEE and the WHO predicted TEE in both men (2208 ± 56 vs. 2915 ± 31 kcal/d (p < 0.05), respectively) and women (1814 ± 42 vs. 2315 ± 21 kcal/d (p < 0.05), respectively) (Figure 2). In both sexes, the WHO equation greatly overestimated TEE, although it was consistent in the overestimation across a broad range of TEEs. No differences were found between DLW measured TEE and DRI predicted TEE for either men (2208 ± 56 vs. 2305 ± 35 kcal/d (ns), respectively) or women (1814 ± 42 vs. 1781 ± 20 kcal/d (ns), respectively).

To determine predictors of TEE in the 8th decade of life, we performed a stepwise multiple regression. To accomplish this, we used 4 different model selection methods including adjusted R^2^, forward selection, backward elimination, and stepwise inclusion techniques. Factors included in the model were age, sex, race, body weight, BMI, SBP, DBP, RMR, percent body fat, and FFM. The alpha-to-enter and alpha-to-stay were set to 0.1 and all potential predictor variables were included in each model. The best model was determined to be the model with the fewest predictors, the lowest mean square error (MSE), and the largest R^2^. Multiple regression analysis was used to model TEE with the predictors obtained from the model selection methods.

Adjusted R^2^, forward selection, backward elimination, and stepwise inclusion all indicated the best model to be one in which age, race, DBP, RMR, and FFM were included with individual F values of 10.73, 3.61, 4.89, 4.09, and 29.60, respectively (intercept = 7.43) and a model F value of 43.43. Finally, in order to confirm the model selection methods, we ran a multiple regression analysis containing age, DBP, RMR, and FFM. Given that age, DBP, RMR, and FFM were included in the model, DBP and race was removed as their p values were p = 0.055 and p = 0.06, respectively. Therefore, the final resulting model contained 3 variables with an adjusted R^2^ = 0.65 (p < 0.0001). Based on the multiple regression model, our results suggest that 65% of the variance in total energy expenditure in late life (e.g. 8th decade) is accounted for by age, RMR, and FFM.

## Discussion

Prior to this study, only one study had been published on energy requirements in individuals in very late life [17]. Further, most studies assessing energy requirements take a cross sectional approach, so no information regarding changes in energy requirements in late life were available. Since decreases in TEE increase obesity risk, understanding the energy requirements in late life and how they change over time is critical [1]. Based on DLW measures of TEE, we found that the energy requirements in very late life (>80 years of age) were 2208 ± 376 kcal/d for men and 1814 ± 337 kcal/d for women. As expected, these TEE values are slightly greater than those reported by Rothenberg et al. [17] who reported TEE to be around 1936 kcal/d and 1506 kcal/d for men and women over 90 years of age, respectively. Further, these values were slightly lower than those reported in studies with subjects ranging from 55–79 years of age. Blanc et al. [16] reported TEEs of 2521 kcal/d (men) and 1885 kcal/d (women) for subjects between the ages of 70–79. Similarly, Carpenter et al. [15] reported TEEs of 2584 kcal/d and 1946 kcal/d in men and women over 55 years of age, respectively. Although the energy requirements decreased in both men and women from the 8th to 9th decade of life in our study (baseline to follow-up), the decrease in TEE was significant in men, but not in women. The average decrease in the energy requirement during the 7-year time span was −274 ± 338 kcal/d in men (p < 0.05) and −77 ± 322 kcal/d in women (ns). Other notable decreases were found in RMR, AEE and PAL in men, but not women.

The repeated measure design captured the average decrease in energy requirements due to aging, but it was also possible to gain some insight into the between individual differences in the rate of the aging effect. Trabulsi et al. [27] examined the precision of DLW measurements of TEE in 24 subjects measured twice within a 2-week period which is a short period that should be free of the influences of aging. They found that the coefficient of variation (CV) in TEE was 5.1% which included a 2.9% analytical variation and a 4.2% physiologic variation. This 5.1% CV is for a single measure thus contributed a 7.1% (5.1x1.4) CV to the change score, which was smaller than that observed in the men (11%) in our study. This suggests that our data represents actual changes in these subjects from baseline to follow-up. Further, we have previously reported the variation in RMR measurements on 2 consecutive days to be 3.0% [28]. This 3.0% CV and 4.2% CV to the change score was also smaller than that which was observed in the men (6.75%) and women (6.89%) from baseline to follow-up.

We wanted to determine whether the changes in TEE from baseline to follow-up could be attributed to changes in RMR, changes in AEE (or PAL), or a combination of the two. Only the men showed a significant decrease in TEE. They also had significant decreases in RMR, AEE, and PAL. The average decrease in RMR was 70 ± 125 kcal/day which represents about 25% of the decrease in TEE. AEE decreased an average of 177 ± 301 kcal/day which accounts for the majority of the decrease in TEE. Therefore, we can conclude that the decreases in TEE in male subjects were mostly due to decreases in AEE rather than RMR. This suggests a priority for promoting physical activity in older persons. Since the female subjects did not show decreases in TEE, it is not surprising that neither RMR nor AEE (or PAL) decreased significantly from baseline to follow-up. It should be noted that women did start at a lower AEE and PAL at baseline, so any possible decline in activity would not be expected to be as great as that observed in men. However, their PAL level was not as low as a very sedentary individual, so it would have been plausible for the women to show a decrease in activity. A decline was simply not observed in the women in this study.

Since we observed decreases in both RMR and AEE in men, we then wanted to determine if specific variables could account for those changes. We set out to establish whether or not changes in FFM could explain the observed changes in RMR, and whether or not changes in body weight could explain the observed changes in AEE. Both RMR and FFM significantly decreased in male subjects from baseline to follow-up. Further, there was a positive correlation between the change in RMR vs. FFM for men (r = 0.49, p < 0.001). Therefore, approximately 49% of the decrease in RMR could be explained by decreases in FFM for men. This highlights the importance of not just increasing physical activity, but also performing more resistance exercise to prevent or slow down loss of muscle mass in aging adults, especially men. However, we acknowledge that this recommendation is only effective if elderly adults are willing and able to perform resistance exercise. The application of this recommendation is questionable since a very small percentage of the adult U.S. population actually meets physical activity guidelines [29] and less than 20% of men and women report strength training two or more times per week [30]. Both AEE and body weight also decreased significantly in men from baseline to follow-up. However, a correlation analysis between changes in AEE vs. changes in body weight did not reach significance (r = 0.24, p = 0.10). Therefore, we cannot conclude that changes in body weight accounted for the changes in AEE that were observed in the male subjects.

In female subjects, both FFM and body weight decreased significantly, but there were no changes in AEE or RMR. This could be due to the fact that the decrease in FFM was relatively small (0.5 kg) and was possibly not a great enough change to alter RMR. Therefore, there was no significant correlation between changes in RMR vs. changes in FFM (r = −0.08, ns) or for changes in AEE vs. changes in body weight in women (r = −0.07, ns). It should be noted that the correlation analyses for both men and women had relatively small sample sizes (n = 47 for men and n = 40 for women). Therefore, it is possible the changes in these variables or total subject number were simply too small to yield significant changes or correlations. However, even with that small sample size, some significant changes and correlations were observed in men. Therefore, based on this data, we believe that elderly women may just not show the same pattern of change in TEE, RMR, and AEE that men do. Finally, it should also be noted that the women in our study did show decreases in AEE and RMR; they just were not statistically significant. It appears that there was a large amount of variation in this data, especially the AEE data in women at follow-up. This large amount of variation could explain the lack of statistically significant declines in women and/or also shows that variation in AEE may be greater in women in the 9th decade of life compared to men. Additionally, the women in this study had lower PALs to begin with compared to the men. This could have impacted the magnitude of decreases in PAL, or lack thereof, in women compared to men. This still indicates that changes in these variables in women are different than men in late life, but it may be partially due to women starting at lower PALs.

As shown above, sex differences of longitudinal change of TEE, RMR, and AEE were found, and the decreasing rate with aging was larger in men than in women. Although its biological reasons are unknown, the results of several previous longitudinal studies may have relevance to this phenomenon. Nakamura and Miyao [31] reported that the rate of biological aging calculated by using 7-year longitudinal data of forced expiratory volume, systolic blood pressure, red blood cells, albumin, and blood urea nitrogen was faster in men than in women. Kimura et al. [32] reported that the rate of physical fitness aging calculated by using 7-year longitudinal data of walking speed, functional reach, one leg stand, vertical jump and grip strength was also faster in men than in women. It would be interesting for future studies to explore the relationship between aging of metabolic aspects and biological or physical functional aspects.

Several prediction equations exist to predict or estimate TEE. As expected, there is some error associated with each of these measures as an estimate of physical activity is required. The DRI and WHO prediction equations have been widely used to estimate energy requirements. However, the WHO equation does not provide an equation for individuals over the age of 60 years. It was unknown how accurately either the DRI or WHO equations could predict TEE in an elderly population in their 8th or 9th decades of life. Only the DRI equation predicted a similar TEE as the measured TEE. In both men and women, the WHO equation significantly over-predicted TEE. This over-prediction occurred with using the lowest level of activity factor associated with the WHO equation (activity factors range from 1.6-2.6; 1.6 was used in this analysis). The Bland-Altman plots indicated that the over-prediction of TEE by the WHO equation occurred at all ranges of energy expenditure while the DRI was accurate at all ranges of energy expenditure. Additionally, the limits of agreement were much wider or greater for the WHO plot indicating worse agreement than that of the DRI plot with DLW measured TEE. Based on these results, it is apparent that the development of an age appropriate WHO equation is necessary for individuals in very late life. Our multiple linear regression analysis indicated that age, RMR, and FFM were the best predictors of TEE in this population. However, obtaining RMR and FFM measurements is difficult to do for the general public, so developing an equation using these variables is likely not clinically feasible.

Very little is known about the energy requirements in very late life. This study provides some initial insight; however, some limitations do exist. It may not be appropriate to extrapolate this data to other populations in the U.S. or worldwide. While this study represents one of the largest longitudinal studies on changes in EE components among older adults, only 27.48% of the initial sample completed the follow-up measurements. Compared to those who did not participate, the follow-up sample had a lower prevalence of cancer, had a greater physical performance score, and spent more time performing physical activity at baseline. We acknowledge that the sample is biased toward those individuals healthy enough to participate in the follow-up study. Consequently, we caution that the results may be skewed toward the healthier subset of the aged and thus are only modestly representative of the age-related changes in EE experienced during aging in late-life. However, it is also possible that these subjects are similar, and therefore, representative to those that are surviving into their 9th decade of life. Importantly, even if these subjects are “healthier” than others of the same age, disease prevalence and the number of diseases affecting at least some of the participants in this study was quite high compared to what one might find in a younger population. Therefore, due to the relatively small number of subjects and the many potential confounding variables (many diseases), we did not adjust variables such as TEE, RMR, AEE, or FFM for any of these potential confounders.

## Conclusion

The energy requirements in the 9th decade of life were 2249 ± 413 kcal/d for men and 1781 ± 315 kcal/d for women. These energy requirements, based on DLW measures of TEE, decreased significantly from baseline to follow-up in men, but not in women. Both RMR and AEE also decreased significantly in men only, and the significant decrease in RMR in men was positively correlated with decreases in FFM. It appears that changes in EE, or lack thereof, in late life differ between men and women. Importantly, however, PAL and AEE levels in women were lower than that of men at baseline. Therefore, the lack of significant decreases in women may reflect their lower starting PALs and a greater opportunity for changes (decreases) in men due to starting at a higher PAL. Finally, while the DRI equation to predict TEE was very comparable to measured TEE (via DLW), the WHO equation greatly over-predicted TEE in our elderly population.

## Competing interests

The authors declare that they have no competing interests.

## Authors’ contributions

All authors contributed to this manuscript. JEE, SC, DCM, ABN, NWG, FT, and TH designed research and conducted research; JAC and CMP analyzed data; JAC and DAS wrote the paper; JAC, TM, CMP, YY, TH, and DAS had primary responsibility for final content. All authors read and approved the final manuscript.
